# Urban Pedestrian Routes’ Accessibility Assessment Using Geographic Information System Processing and Deep Learning-Based Object Detection

**DOI:** 10.3390/s24113667

**Published:** 2024-06-05

**Authors:** Tomás E. Martínez-Chao, Agustín Menéndez-Díaz, Silverio García-Cortés, Pierpaolo D’Agostino

**Affiliations:** 1 Department of Civil, Building and Environmental Engineering, University of Naples “Federico II”, 80125 Naples, Italy; tomasenrique.martinezchao@unina.it (T.E.M.-C.); pierpaolo.dagostino@unina.it (P.D.); 2 Department of Construction and Manufacturing Engineering, University of Oviedo, 33004 Oviedo, Spain; 3 Department of Mining Exploitation and Prospecting, University of Oviedo, 33004 Oviedo, Spain; sgcortes@uniovi.es

**Keywords:** inclusiveness, geographic information system (GIS), pedestrian crossing, deep learning, wheelchair-friendly routes, inertial sensors

## Abstract

The need to establish safe, accessible, and inclusive pedestrian routes is considered one of the European Union’s main priorities. We have developed a method of assessing pedestrian mobility in the surroundings of urban public buildings to evaluate the level of accessibility and inclusion, especially for people with reduced mobility. In the first stage of assessment, artificial intelligence algorithms were used to identify pedestrian crossings and the precise geographical location was determined by deep learning-based object detection with satellite or aerial orthoimagery. In the second stage, Geographic Information System techniques were used to create network models. This approach enabled the verification of the level of accessibility for wheelchair users in the selected study area and the identification of the most suitable route for wheelchair transit between two points of interest. The data obtained were verified using inertial sensors to corroborate the horizontal continuity of the routes. The study findings are of direct benefit to the users of these routes and are also valuable for the entities responsible for ensuring and maintaining the accessibility of pedestrian routes.

## 1. Introduction

Functional diversity and physical disability should not be barriers to citizens’ social interactions in their environments, considering that the use of communal spaces contributes to human well-being and safety [1,2]. Accessibility for all must be a priority, and interventions must be made to achieve this goal. Access points (streets, pavements, and building surroundings) also form part of urban spaces and must comply with safety and accessibility measures [3]. The European Union aims to develop inclusive, safe, accessible, and sustainable transport systems and has created numerous programs promoting mobility as a universal, essential right [4]. Considering that currently more than 80 million Europeans suffer from a long-term disability [5], ensuring inclusive mobility and participation in society would help to reduce the exclusion experienced by these individuals. Other strategies of Sustainable Mobility (SM) developed by the European Commission, as outlined in the European Green Deal [6], promote people-centred mobility and aim to make transport infrastructure and services affordable and accessible to all passengers in all regions.

**Figure 1:**
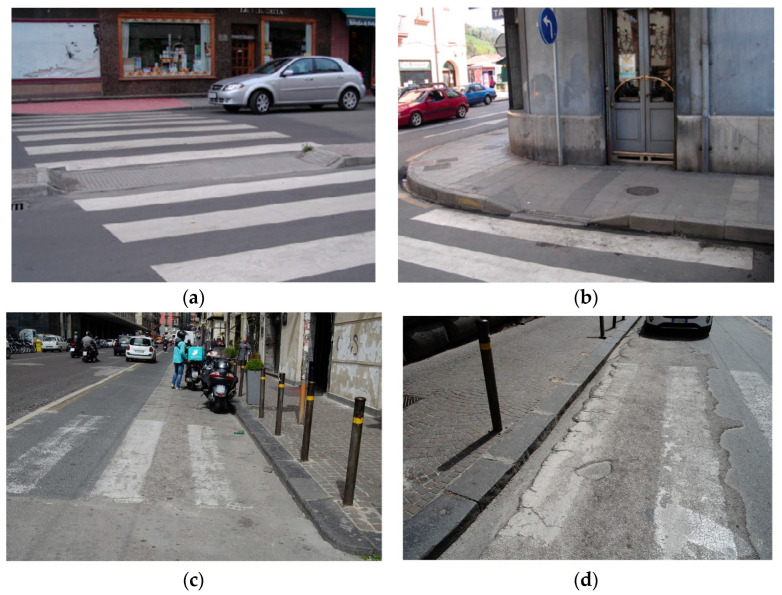
Barriers affecting mobility for pedestrians: (**a**) zebra crossings with different pavement levels, (**b**) curbs with faulty ramps, (**c**) gutter and structural barriers, and (**d**) areas with very deteriorated pavement. Urban routes (**a**,**b**) in Mieres, Spain, and (**c**,**d**) in Naples, Italy.

These strategies recall a quote by Janette Sadik-Khan, Commissioner of Transportation in New York (2002–2013): “Streets are a means to get from one place to another, but they are also destinations in themselves. For many years, we have designed them only for cars and forgotten all the ways they can be used. They are for pedestrians, not for cars” [7] While streets are primarily intended for vehicular traffic, they also incorporate structures designed for pedestrians, such as pedestrian crossings, which facilitate the safe passage of people across the streets. Like any road infrastructure, pedestrian crossings must adhere to various technical characteristics to ensure their functionality [8,9]. Unfortunately, many pedestrian crossings are constructed without considering the need for an inclusive passage for everyone (see Figure 1), especially people who need to use wheelchairs [10]. Other types of functional limitations, such as visual and auditory impairments, which are not part of this research, are also important [11,12,13].

The new technologies and computer resources available at present contribute, to some extent, to simplifying people’s lives. Today, more than ever, artificial intelligence algorithms are used in daily life. In this study, we used machine learning algorithms to identify and assess the accessibility and inclusivity of pedestrian crossings. Simultaneously, we conducted a geospatial analysis to identify the optimal routes for wheelchair users by including a graphical analysis of the results derived from the classification and location of pedestrian crossings. This approach enables informed decision-making when establishing routes, ensuring safe, inclusive mobility. It also serves as an informative tool for the entities responsible for the planning and restoration of public roads, facilitating timely intervention where these issues exist.

**Figure 2:**
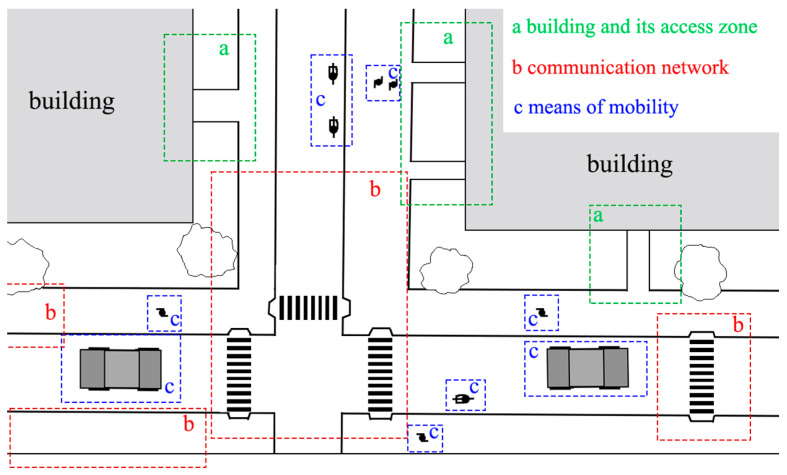
Elements affecting the mobility around a building in an urban area.

The urban fabric that we navigate within cities coexists with buildings and communication routes that facilitate mobility but also include significant barriers, thus influencing mobility. To address this, we must consider the following elements (see Figure 2):

(a) The building and its access zone. The access zone includes the entrances and exits to a building that enable movement without structural barriers and facilitate the passage of pedestrians or individuals in wheelchairs. A single building can have one or multiple access zones. If it serves a different purpose or was established for other utilitarian reasons, the access zone can be considered an aggregation of contiguous zones of different natures (loading and unloading zones, vehicle boarding zones, etc.).
(b) The communication network. The building extends its access zones towards the surrounding communication network, which is primarily composed of roads and pathways. This communication network separates each building access zone. It should be understood as a series of communication routes (roads, railways, paths, roundabouts, etc.). Communication, in a broad sense, establishes the connection and mobility between the access zones of different buildings (either adjacent buildings or those separated by a complex urban fabric). Each route will have a communication carriageway, delimited on the left and right by sidewalks extending to serve other accessibility zones in the urban fabric. Zebra crossings serve as connective elements, enabling the axis of this communication network to be crossed to facilitate access to both sides of the axis, primarily enhancing pedestrian mobility. In modern cities, zebra crossings are also designed with universal accessibility criteria for use by individuals with mobility issues, especially individuals using wheelchairs, walkers, crutches, etc. A pedestrian pathway is different in nature in that its central axis is designed to enhance the mobility of pedestrians and wheelchairs, representing the most flexible way to channel mobility from one accessibility zone to another safely and without any barriers. A bike lane is similar to a road but has a different access point from that of a road, as it may or may not be shared with the pedestrian pathway, depending on its design and integration in other communication networks.
(c) The means of mobility. In this case, we must consider the means used to navigate within the network using specific elements (vehicles, pedestrians, wheelchairs, bicycles, scooters, etc.). Individuals may use a combination of various means of mobility; in some cases, limitations may restrict them to using only one. In any case, the communication network should ensure mobility for individuals using one or more methods to move from one building to another.

A pedestrian route can be analysed considering three scales of accessibility: urban, proximity, and building. Urban routes are small-scale routes and include various blocks and streets, connecting one point of interest to another (home, work, public buildings, urban areas, etc.). Proximity routes are intermediate-scale routes that consist of areas surrounding and adjacent to buildings or urban areas. Finally, building access routes include the detailed internal paths within each structure, building, or area of interest, considering mobility through corridors, rooms, spaces, ramps, and elevators.

Each type of scale includes elements that influence accessibility analyses and can be used to assess the conservation and inclusivity they exhibit. Initially, a preliminary literature review was conducted regarding the implementation of recognition algorithms and the use of GIS in monitoring road infrastructures, particularly those adapted for pedestrian traffic, specifying the scale of accessibility to which they are applied (see Table 1).

Among the studies analysed in the previous table, we can observe the use of artificial intelligence algorithms applied to the recognition of structural elements of roadways. This is evident in the work presented by Weld G. et al. [16] and K. et al. [19]. In both cases, panoramic images from Google Street View (GSV) were used for the recognition and labelling of pedestrian ramps. In the first study, a modified residual neural network (ResNet) was employed, which improved the labelling of objects in the images. The second study used computer vision algorithms and deep learning, which, together with GSV images, yielded optimal results in recognising pedestrian ramps. The work conducted by Kaya Ö. et al. [20] utilised R-CNN and YOLOv7 network models to detect zebra crossings from images obtained from a database. In all three studies, the detection of elements was carried out using ground-level images, which limited the detection of objects such as zebra crossings over large areas. We addressed these issues in our work by using aerial orthoimages for the detection of zebra crossings in more extensive areas.

We also analysed studies that used GIS to measure the level of accessibility of buildings [17]; in this case, the authors focused on the development of a new metric to value the relative accessibility between routes but their data information capture system was mainly manual. This is like the studies by Beale L. et al. [18] and Pérez-delHoyo R. et al. [22], where data collection was carried out manually to create GIS databases. This limits the automation of analyses, a factor that we aim to address in our work. In addition, we examined other studies focusing on the management of accessibility problems in urban environments. The study by K. Karamitov et al. [14] evaluates accessibility at the macro level in the city of Sofia, so it does not consider the accessibility of routes and their level of adaptation for wheelchair users.

All these studies, whether they employed AI or GIS, aim to improve accessibility conditions for users in general. Our work intends to incorporate aspects of these studies and combine them with new methodologies, primarily targeting wheelchair users, with a higher level of detail, including the use of inertial sensors for deformation control.

After analysing the proximity type (building surroundings) of the access routes [24], we developed a method considering urban routes. The analysis of elements of particular importance includes pavements and pedestrian crossings and their access points (ramps). To analyse the conditions of the elements in each route, the exact location of these elements must be determined, and the level of accessibility and inclusion should subsequently be assessed.

In this article, we first describe the most characteristic elements of our research and the initial hypotheses (Section 2). We explain the method used to carry out the analysis in three phases in Section 3. In Phases 1 and 2, we define how the input data were structured and we demonstrate that the processing involved identifying pedestrian crossings and defining the mobility routes along pavements. Finally, in Section 4, we explain how the results were analysed (in phase 3). To illustrate the operationality of each phase, the application of the proposed method to a specific case (an urban environment) is discussed, and the results obtained in each phase are presented in this section (Section 4). Finally, we present the key conclusions of the study in Section 5.

## 2. Types of Pedestrian Crossings and Main Characteristics of Ramps

Before undertaking urban mobility studies, it is essential to understand the main characteristics of the structural elements of roads, especially those that play a significant role in the mobility of disabled individuals. These elements include ramps and pedestrian crossings.

The design and functionality of pedestrian crossings and access ramps vary depending on the surrounding conditions, the intended functionality, and the area in which they are located. The presence of pedestrian crossings can be indicated in different ways, and in some cases, there may be no indication, which poses a risk to pedestrians.

There are a wide variety of types of pedestrian crossings and access ramps, all of which must adhere to specific parameters regarding width, incline, and location [25,26,27].

Some pedestrian crossings are indicated by vertical signs or traffic lights, the latter of which are generally the safest indicators. Other types include raised pedestrian crossings with speed bumps, which compel vehicles to slow down. Smart pedestrian crossings illuminate the route when pedestrians are ready to cross, and 3D pedestrian crossings are designed with optical effects to enhance detection by vehicles. The final types include yellow pedestrian crossings, which are mainly intended for temporary use, and pedestrian crossings combined with lanes for cyclists and, in some cases, for two-wheeled motor vehicles.

European and local standards in different countries [28,29,30], aimed at promoting safe and inclusive mobility, outline the minimum characteristics that pedestrian routes must meet. These standards are divided into three main groups to ensure the minimum conditions for wheelchair accessibility: the width, slope, and gradient of the path/route.

**Figure 3:**
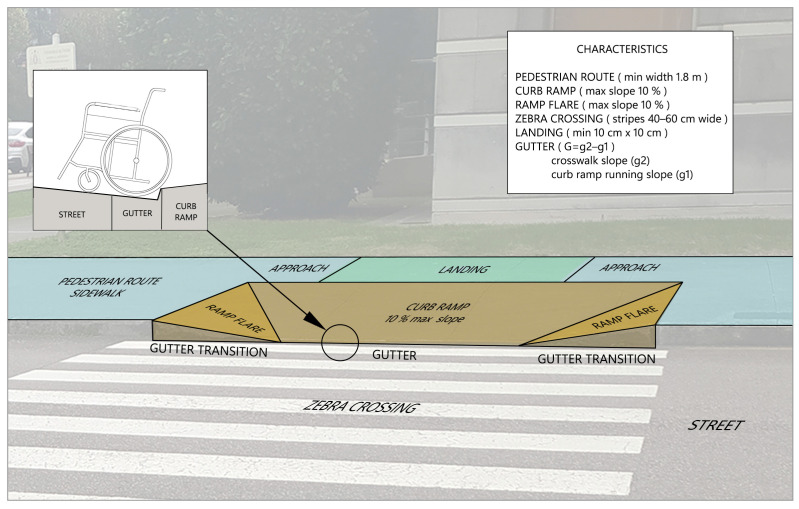
Principal elements and geometrical characteristics of a pedestrian crossing.

As well as a variety of pedestrian crossings, there are many types of access ramps. However, all must comply with some essential minimum characteristics to ensure accessibility for all [31,32,33] (see Figure 3). The elements of pedestrian crossings and their geometrics characteristics are as follows:

(a) Pedestrian routes: These must have a minimum free width of 1.50 m, with occasional narrower parts (approach) of at least 1.20 m. They should have a clear height, without obstacles, of 2.20 m. The longitudinal slope should be less than 8% and transverse slopes should be less than 2%. The surface must not have any deformations that include changes in level of more than 4 mm.
(b) Zebra crossings: The stripes should always be positioned facing each other. If this is not possible, a tactile guiding stripe of width 5 cm and height 6 mm should be installed on both sides. If the crossing, due to its length, has two parts with an intermediate stop, the island should have minimum dimensions allowing the inscription of a circle with a diameter of 1.50 m. The width of the zebra crossing should be at least 1.50 m.
(c) Curb ramps: These should be placed outside the usual line of pedestrian flow, without obstructing the minimum path width of 0.90 m. The maximum slope should be 10%, with a minimum width of 0.90 m and a recommended width of 1.20 m.
(d) Ramp flares: The slope should not be greater than 10%, as is true for the curb ramps.
(e) Landings: These are created for resting, manoeuvring, and to prevent vehicles travelling at excessive speeds on the ramps. They should be at least 1.20 m in length and be the same width as the ramp.

## 3. Methodology

**Figure 4:**
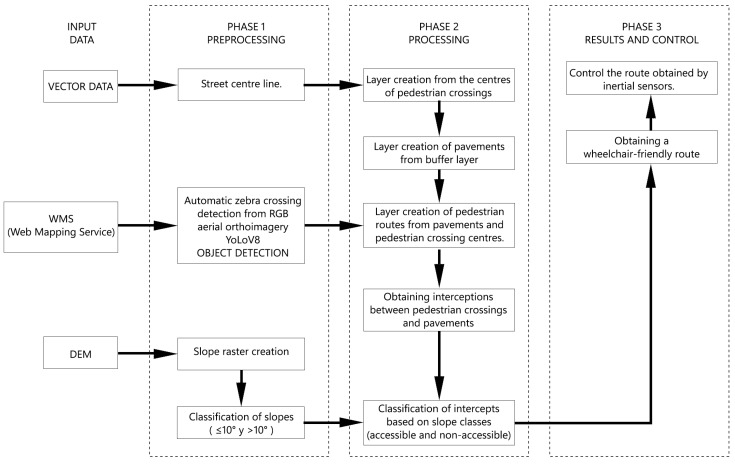
Methodological process.

In order to implement the study, we developed a method in three phases (Figure 4); different types of data were obtained and used in each phase.

Phase 1: In the pre-processing phase, input data were prepared for subsequent geospatial analysis. Lines corresponding to the street axes and their main characteristics (width, typology, name, etc.) were extracted from vector data taken from publicly available open data from OpenStreetMap [34] for the study area. In this initial phase of the methodology, the spatial location of pedestrian crossings in the study area was determined by using an AI algorithm with orthoimages retrieved from official Spanish cartography web services [35]. The absolute coordinates of the centre of each pedestrian crossing were then obtained. Finally, a slope raster was created from the DEM developed with a medium resolution of 3 cm/pixel. The slope raster was classified as slopes less than or equal to 10° and slopes greater than 10°.
Phase 2: In this phase, the goal was to determine all the connecting elements between two points (IP, initial point, and FP, final point) in the study area and to classify pedestrian crossings based on their level of accessibility.
Phase 3: The most suitable routes for people in wheelchairs were determined and accessibility was monitored using inertial sensors. This phase was created on the basis of the explanation provided in one of our earlier studies [24] to determine the most suitable route.

To apply this in a real environment, one of the busiest areas around the Escuela Politécnica de Mieres (EPM), University of Oviedo, Spain) was chosen for study. The main bus station was selected as the initial point (IP), and the university entrance was chosen as the final point (FP). The aim of selecting this area was to assess the level of accessibility and inclusion in the EPM buildings.

### 3.1. Phase 1: Preprocessing

First, the areas corresponding to vehicle transport streets were imported into QGIS 3.28.8 [36]. These were represented by a vector layer of lines for the centre axes of vehicular lanes (SC), downloaded from OpenStreetMap [34] and imported in the absolute coordinate system (EPSG 25830). The vector data corresponding to the study area were extracted, and additional information regarding the approximate width of each street was added.

**Figure 5:**
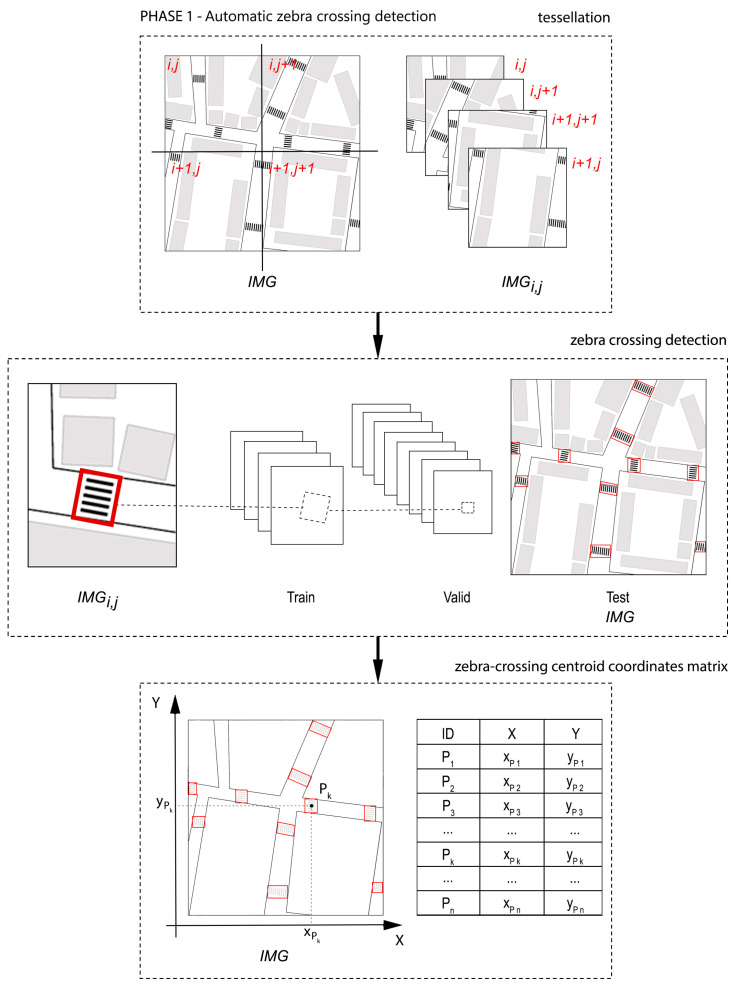
Automatic detection of zebra crossings and identification of the centres of the crossings.

**Figure 6:**
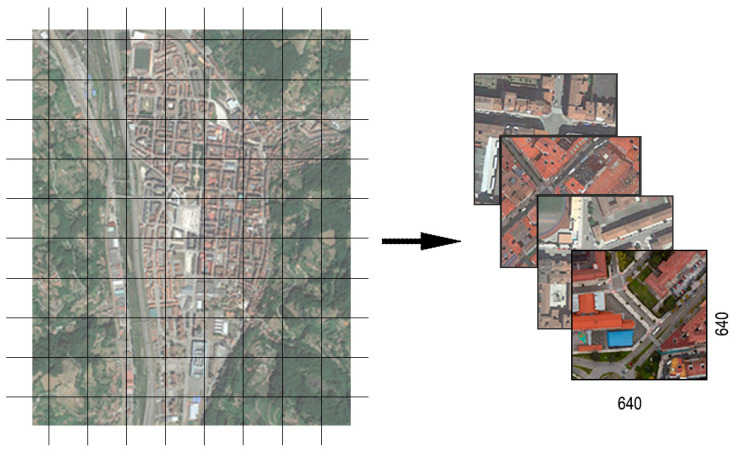
Satellite image tessellation of Mieres city (Spain).

Subsequently, the automatic detection of zebra crossings was carried out using RGB aerial orthoimages obtained through an online request to the Web Map Service (WMS) (Figure 5) from Spanish PNOA servers. At this stage of the process, the YOLO architecture was trained. For this study, a simple app was coded to allow the user to define the study area by enclosing it in a rectangular polygon on a viewer, with the corners stored. A request was then made to the appropriate WMS to return the orthoimage tiles of the area with the maximum available resolution and their georeferencing file. An example of the whole-area image can be seen in Figure 6, at a reduced size.

During this training process, we divided the requested whole image from the WMS into a set of orthoimage tiles to train, validate, and test the object detection architecture on RGB images. The georeferencing file corresponding to each tile was constructed as a text file with a jgw extension, using the same name as the georeferenced image. The values in this file are easy to generate from the GSD values of 0.25m and the limits of each tile in absolute coordinates (in our case, in CRS: EPSG 25830, meaning UTM 30N ETRS89). The 455 images of the dataset were obtained from various Spanish cities located in the Principality of Asturias (specifically Mieres, Oviedo, and Gijón). The dataset was manually annotated.

This architecture was trained with this dataset and then learned to automatically detect this type of horizontal signalling (zebra crossings). The products of this detection are the positions (in local coordinates from each tile) of the automatically obtained zebra crossings that, using the georeferencing metadata, are backtransformed to the absolute coordinates of the existing zebra crossings. These coordinates were stored to build possible routes and to ensure the selection of the optimal accessible route in subsequent stages of the process.

**Figure 7:**
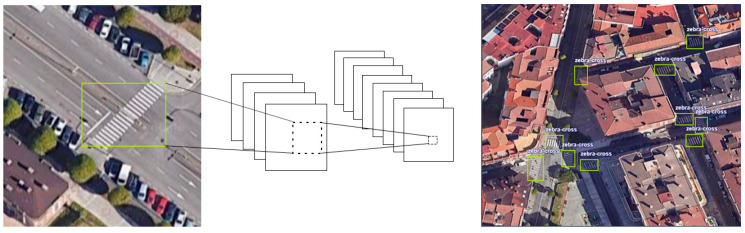
Application of YOLO object detection algorithm in mosaics.

The You Only Look Once (YOLO) object detection algorithm was applied to the tiles (Figure 7). This algorithm, developed by Joseph Redmon and Santosh Divvala at the University of Washington [37], relies on computer vision and deep learning techniques. Unlike other algorithms that involve multiple stages and regions, YOLO focuses on detecting objects in a single pass through the convolutional neural network. It divides an image into a grid, predicting bounding boxes and object classes in each grid cell in a single inference. This feature significantly improves the speed of real-time object detection. Since its creation in 2015, more efficient and faster versions have been developed for object detection. For this study, one of the latest versions was YOLOv8, developed by Ultralytics [38,39], which builds on the success of previous YOLO versions and incorporates new features to enhance performance and flexibility.

**Figure 8:**
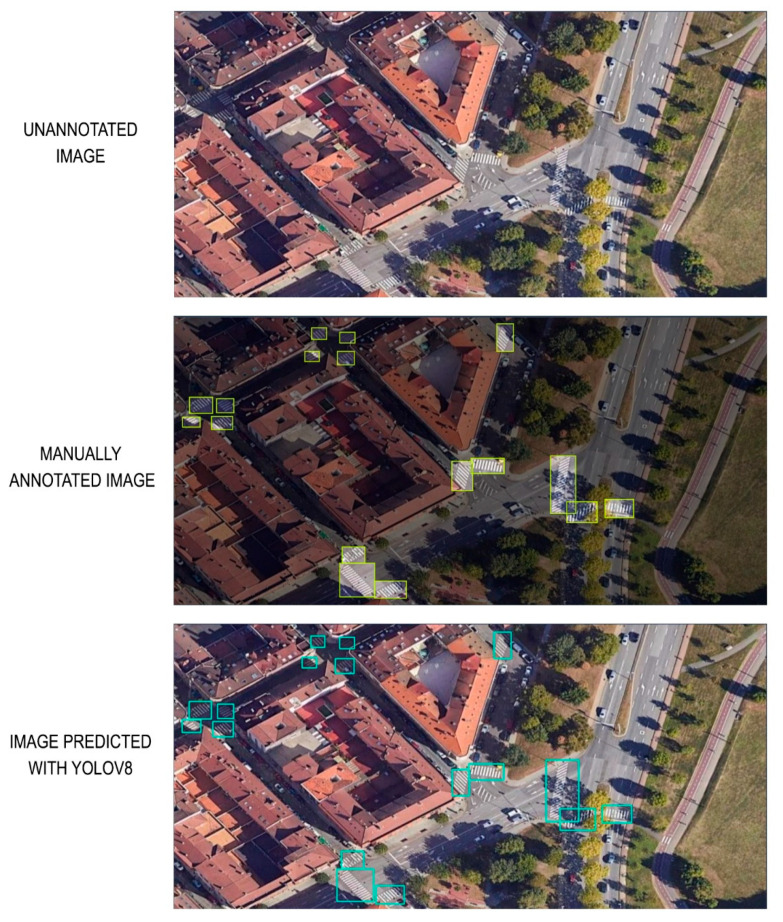
Examples of training images and zebra crossing detection results.

This image set was manually annotated to train the object detection architecture, marking the centres of real zebra crossings and a bounding box around the markings. Subsequently, 90% of the images were selected for training (Figure 8). Although the average number of zebra crossings per image in the entire dataset was 5.6, data augmentation was performed using basic transformations that do not involve geometric distortions, such as flips (horizontal and vertical reflections) and 90° rotations. This results in a data augmentation factor of three per sample. The dataset contains 2562 annotations of zebra crossings before data augmentation.

In Figure 8, we present a histogram of the number of images per intervals at which zebra crossings appear in the area. These images have a wide representativeness in terms of the number of zebra crossings. There are images with one zebra crossing and in some cases, up to 2–6 zebra crossings can be found. The largest number of images is concentrated in those areas containing between 2 and 10 crosswalks, which are logically at the junctions of two or three streets.

In the final part of Phase 1, we proceeded to import an in-house-produced Digital Elevation Model (DEM) in raster format, with a resolution of 3 cm/pixel. This resolution enables the identification of vertical variations/inconsistencies in the terrain that are challenging for wheelchair users. This DEM raster is then transformed into a Slope raster, which was classified based on the percentage of slope (class_1 ≤ 10% and class_2 > 10%).

### 3.2. Phase 2: Processing

**Figure 9:**
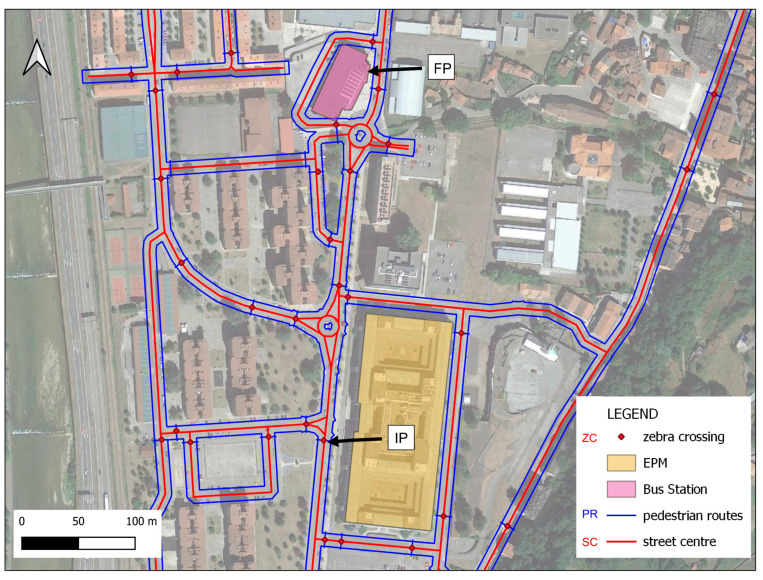
Map showing pedestrian crossings.

A geospatial analysis of the study area was conducted with the data obtained in Phase 1. Initially, using all these data, the elements connecting the Polytechnic School of Mieres EPM (IP: Initial Point) and the Bus Station (FP: Final Point) were identified (see Figure 9). These elements correspond to routes adapted for pedestrian traffic.

Taking the starting data of the street centre axis (SC denoted in red) of each street and the centre of the zebra crossing of the JSON file, a buffer was obtained on both sides of the street axis corresponding to the width of each street plus 3.5 + 1.0 m (3.5 is the normal width of a lane in this area, and an additional meter is considered for the wheelchair). This allowed us to identify the pedestrian routes (PR denoted in blue). To connect one PR to another, a line layer was created, connecting the nearest points of the PR while passing through the centre of the zebra crossing ZC (denoted in red).

Profiles corresponding to the pedestrian crossings in the study area were obtained through very spatially reduced photogrammetric surveys as a means of controlling the slope changes in each. A point layer of ZC was categorized in relation to the slope classification. The following categories were assigned: Not Accessible (NAC) for slopes > 10% and Accessible (AC) for slopes ≤ 10%. The ZC lines including any NAC points were extracted into a new line layer (NA). The other ZC lines were joined with the PR lines, creating a single vector layer of lines corresponding to Pedestrian Routes (PR) adapted for wheelchair users.

### 3.3. Phase 3: Optimal Route Selection

As previously mentioned, the final phase aimed to determine the optimal route for wheelchair users. To achieve this goal, the shortest route between the IP and FP was first determined. Considering that the previous phase did not account for inaccessible pedestrian crossings when creating the PR line layer, this layer was used as the foundation. Using the Network Analysis/Shortest Path (point to point) plugin of QGIS, the sequence of branches that allowed for the shortest connection between the IP and FP points was analysed. For this analysis, the methodology introduced in [24] was used.

## 4. Case Study Results

**Figure 10:**
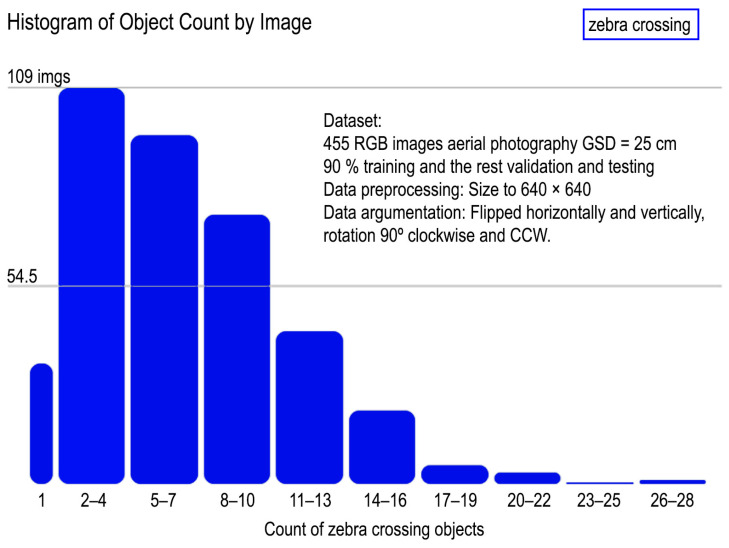
Histogram of zebra crossing counts by image number (for example, 109 images contain between two and four zebra crossing annotations).

In order to illustrate the results of the developed methodology, we analysed the study carried out in the southern area of the city of Mieres, in the surroundings of the university campus. The previously presented annotated image dataset was used. See Figure 10.

A YOLO V8 architecture was trained using the generated dataset. The confidence level reflects the probability that the box contains an object and how precise the box’s dimensions are. Simultaneously, YOLO also predicts the class probabilities for each bounding box position. To filter redundant (very close) or low confidence bounding boxes, it uses the confidence threshold and non-maximum suppression. The result is a combination of bounding boxes and object classes (“zebra cross”-“no zebra cross” in this case) for the detected objects in the image.

**Figure 11:**
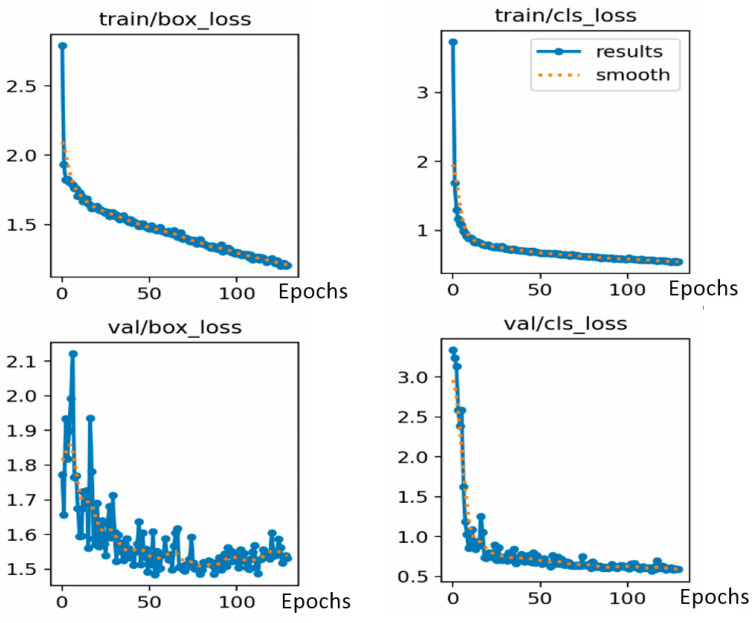
Iterative changes in loss function. By row: “val” refers to the validation set and “train” to the training dataset. By column: “cls_loss” refers to the class of probability loss function and “box_loss” means bounding box position loss function.

**Figure 12:**
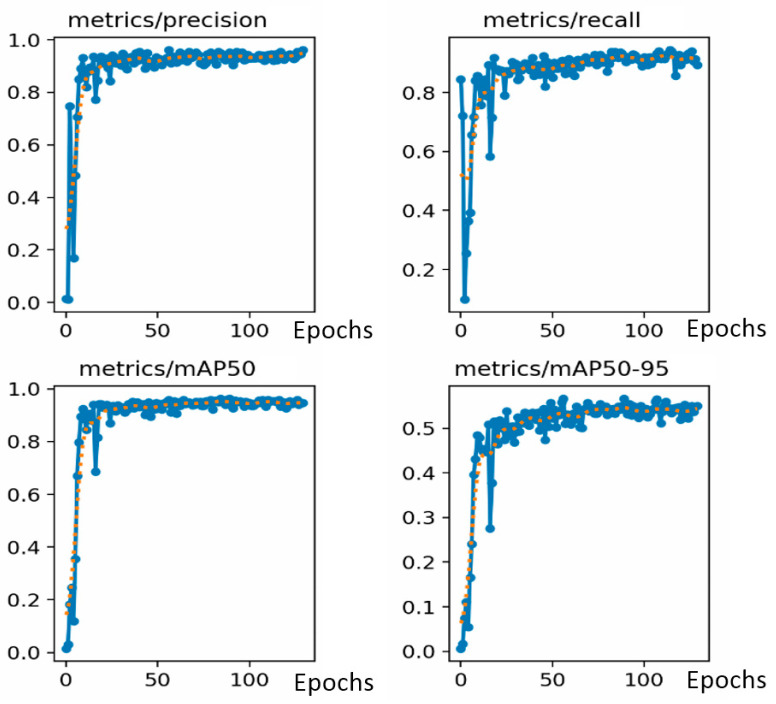
Basic metric evolution (precision, recall, and mAP50-95%) for the Yolov8 object training process through the iterative process (blue color is the result value, and the orange points are the smoothed version of the training metrics).

Changes in loss functions for class and bounding box positions over iterations can be seen in Figure 11. The mean average precision values obtained during training with our dataset are shown in Figure 12. The iterative process is usually stopped before reaching the maximum number of epochs allowed if the convergence of the loss functions is reached after a lower number of epochs. In our case, the process stopped after approximately 130 iterations. The mean average precision values obtained during training with our data set are shown below, along with the changes over 130 iterations. The changes that occurred throughout the iterative process of the loss function in the box, class, and object boundaries are also shown.

In Figure 11, the labels in the x-axis are epoch numbers and for loss graphics, the y-axis labels are unitless and show the magnitude of the accumulated loss error function.

In Figure 12, labels in the x-axis are epoch numbers and the labels in the y-axis are the average precision values for loss graphics (from 0 to 1).

**Figure 13:**
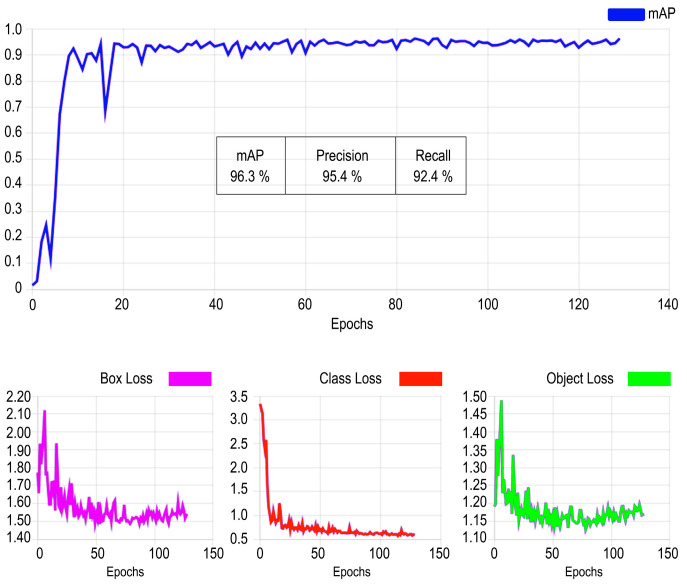
Results of the analysis: Medium Average Precision, precision and recall.

The analysis results yielded a Medium Average Precision of 96.3%, precision of 95.4%, and recall of 92.4% (see Figure 13). Precision refers to the network’s ability to avoid false predictions. Recall provides information about the network’s performance in detecting the maximum possible number of objects. The mean average precision (mAP) indicates the overall behaviour of the network during the detection process. mAP50-95 is the average of the mean average precision for different Intersection over Union (IoU) thresholds, indicating the performance of the detector at different intersection levels (ranging from 50% to 95%). An IoU of 50% means that the overlap between the predicted bounding box and the ground truth bounding box is only of 50% of the total area. When the IoU is 100%, the coincidence between the ground truth bounding box and the detected truth is complete. A high mAP 50%-95% metric value means that the model will perform well not only at a single IoU threshold but across a spectrum of values, making it robust to various detection scenarios, algorithms, and datasets.

**Figure 14:**
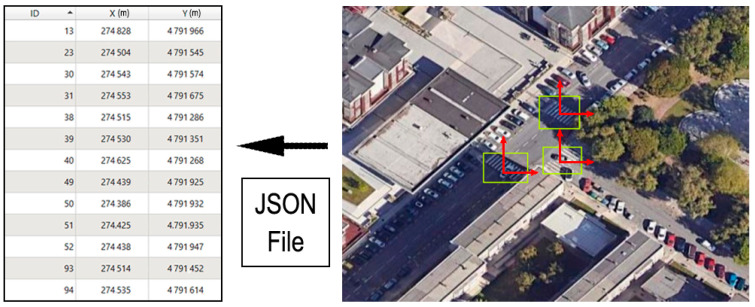
Detection of the centres of zebra crossings (in red color the local coordinate system and in yellow the zebra crossing detected).

The zebra crossings that were detected are returned by the YoloV8 implementation in the form of a JSON file for each inference on an image. The structure of this file and the information it contains for a single zebra crossing can be seen in Figure 14. The output of the detection algorithm is presented in local coordinates for each image. The final step of this phase involves obtaining the absolute coordinates (EPSG 25830) of the centre of each of the identified zebra crossings using the jgw world file generated for each aerial orthophoto tile and the prj file specifying its CRS. This generates a vector file in JSON format containing all the crossings from the image used for detection. This allows us to link the output of the object detection process with the other tasks to find the optimal route.

**Figure 15:**
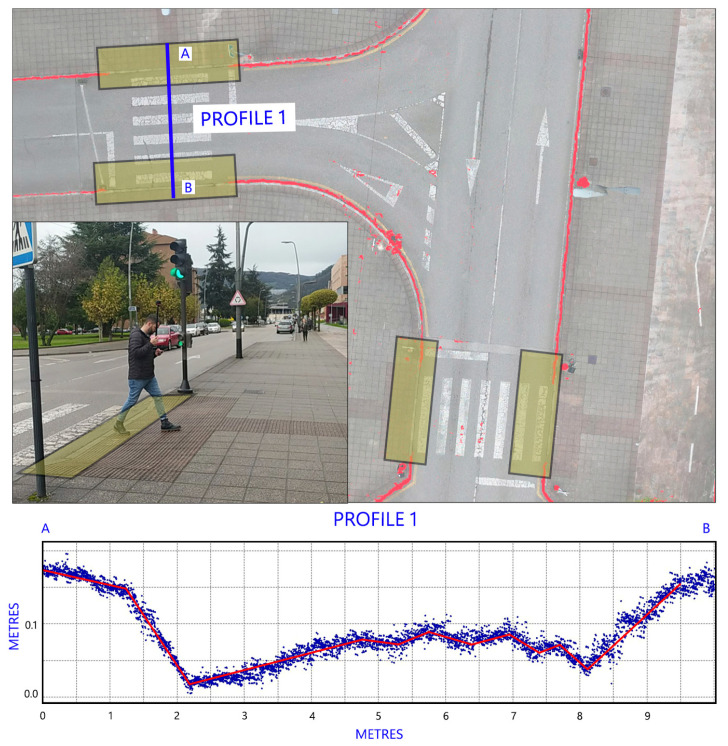
Section AB of one of the accessible zebra crossings (AC) that should be considered when analysing mobility.

**Figure 16:**
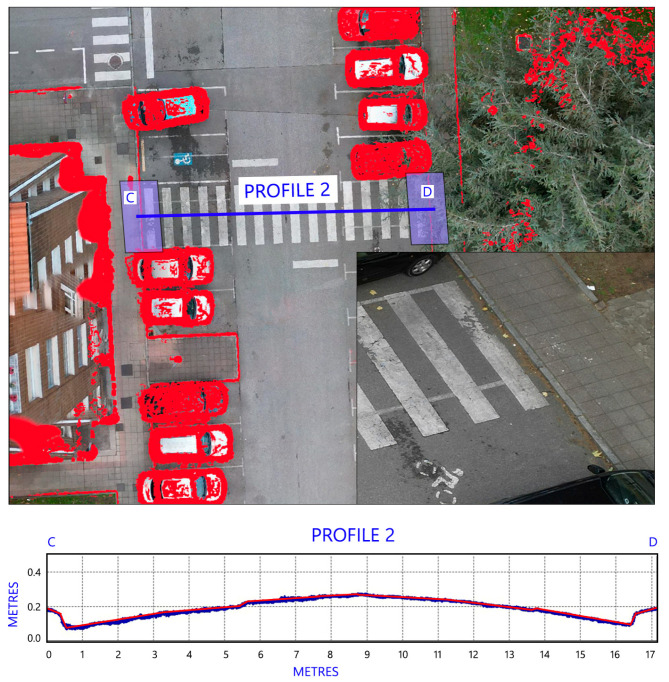
Section CD of one of the non-accessible zebra crossings (NAC) that should be considered when analysing mobility.

As can be seen in section AB (see Figure 15), we have an accessible jump because, in the area near the curb ramp, there is a sloping surface that allows pedestrians to gradually overcome the obstacle. This section corresponds to an AC zebra crossing. In section CD (see Figure 16), the change in inclinations is greater. At the ends of the pavement there are jumps of about 0.15 cm, but this occurs at a horizontal distance of 0.4 m, which is a clear obstacle to mobility using wheelchairs. This section corresponds to an NAC zebra crossing.

**Figure 17:**
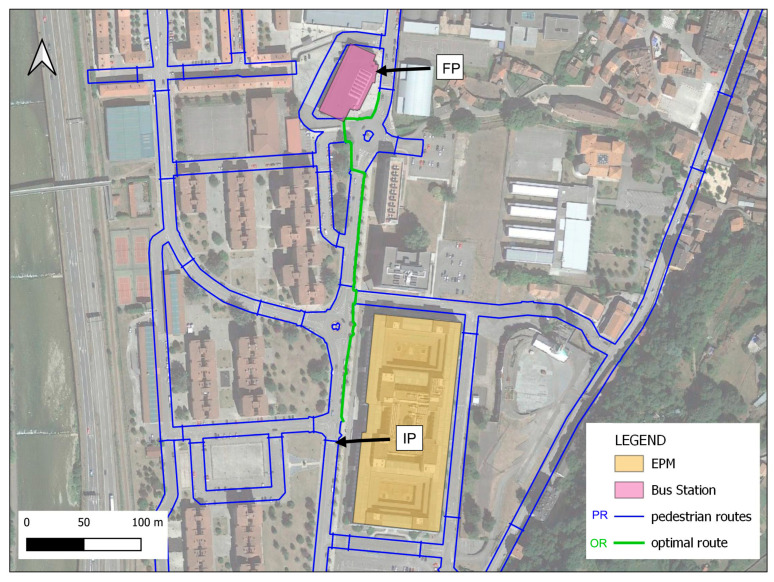
Map showing the optimal route for wheelchair users.

This type of sectional analysis was carried out for all zebra crossings, finally obtaining ACs as valid traversable crossings and discarding impassable NACs. Finally, the methodology indicated in Phase 3 was used to find the shortest route between points PI and PF (see Figure 17). In the Figure, all the lines that are accessible with a wheelchair appear in blue, and in green, the route that allows you to travel the shortest possible distance is displayed. This optimal route (OR, shown in green colour) in terms of distance and accessibility was determined using the Network Analysis/Shortest Path (point to point) plugin of QGIS [40].

**Figure 18:**
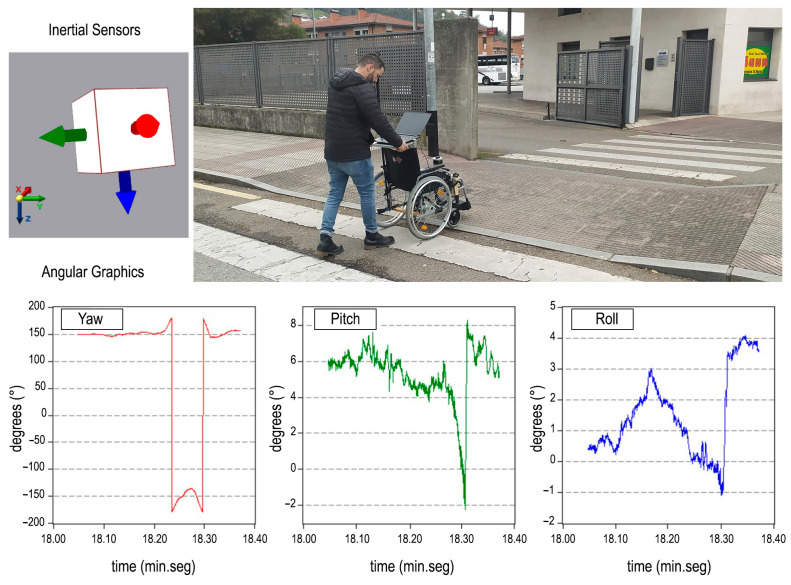
Monitoring the optimal routes by using inertial sensors.

Once the shortest route with the best accessibility conditions was determined, the entire selected route was monitored using inertial sensors (see Figure 18). For this purpose, an adapted wheelchair fitted with an VN-200 inertial sensor was used (see [24]), and a weight of 60 kg was added to simulate a real scenario. The inertial sensor provided data related to turns in each direction (YAW, PITCH, ROLL), enabling an assessment of the complexity of the route and the efficiency of the analyses [24].

## 5. Discussion and Conclusions

Below, we discuss some of the advantages and disadvantages of the developed method.

Among the advantages, we can highlight the following:

- The methodology makes it possible to study a wide area of an urban environment using information available in the open spatial databases of city streets.
- It allows for the rapid identification of zebra crossings and can determine whether the zebra crossing is accessible or not in a semi-automatic way.
- The method can establish, using minimum distance criteria and comfort measurements, the most accessible route between two points for analysis.
- The methodology is good in areas where zebra crossings are homogeneous and well maintained (well preserved and free of obstacles).

Some drawbacks have also been identified:

- If the surface of the zebra crossing has significant irregularities, and it does not conform to the typology of common zebra crossings, the interpretation of the DEM becomes significantly complicated, requiring the user to analyse the zebra crossing manually.
- The methodology can be used in urban areas of large cities, but in many cases in urban parts of rural areas, reliable and up-to-date photographic databases are not available.
- In many cases, data collection using inertial sensors is also hindered by the presence of traffic or adverse weather conditions, which distort the measurements made.

Considering all these aspects, we can conclude that the correct management of road infrastructure, especially of pedestrian roads, is essential from the initial stage of construction. Inclusion and accessibility are also key considerations when constructing and restoring pedestrian routes. This study aimed to consider existing infrastructure and intelligently distribute interventions, prioritizing the most important areas to ensure continuous and inclusive mobility along the entire route. The findings lay the groundwork for addressing the special needs of different users. The use of Artificial Intelligence and GIS algorithms generates real-time, highly accurate, geo-referenced data, representing an important advancement in our mission to create inclusive, safe, resilient, and sustainable cities. This study has also demonstrated that monitoring pedestrian crossings based on geometric criteria produces favourable results. However, the geometric data should be verified by data recorded with inertial sensors to improve the reliability of the results. Finally, the developed methodology produces valuable information for the local authorities responsible for maintaining and supervising such services in large urban areas.

## Figures and Tables

**Table 1:** Mobility analysis at different scales.

Reference	Scale of Accessibility	Use of AI Algorithms	Use of GIS Techniques
[14]	urban	no	yes
[15]	proximity	no	yes
[16]	urban	yes	no
[17]	buildings	no	no
[18]	proximity	no	yes
[19]	proximity	yes	yes
[20]	proximity	yes	no
[21]	urban	no	yes
[22]	urban	no	yes
[23]	proximity	no	yes

## Data Availability

Data are contained with the article.
